# Supplementary material for: Sae2 controls Mre11 endo- and exonuclease activities by different mechanisms
Source: Nat Commun. 2024 Aug 22;15:7221. doi: 10.1038/s41467-024-51493-5 (PMC11341764; doi:10.1038/s41467-024-51493-5)
Supplement: Supplementary file 1 — Supplementary Information [file 41467_2024_51493_MOESM1_ESM.pdf]

Supplementary Table

Table S1 - Sequence of the oligonucleotides used for the preparation of DNA substrates.

Name	Sequence (5’ to 3’) - underlined bold **T** indicate the position of biotinylated dT nucleotides
PC210	G**T**AAGTGCCGCGGTGCGGGTGCCAGGGCGTGCCCTTGGGCTCCCCGGGCGCGTACTCCACCTCATGCA**T**C
PC211	GA**T**GCATGAGGTGGAGTACGCGCCCGGGGAGCCCAAGGGCACGCCCTGGCACCCGCACCGCGGCACT**T**AC
X12-3	GACGTCATAGACGATTACATTGCTAGGACATGCTGTCTAGAGACTATCGC
X12-4C	GCGATAGTCTCTAGACAGCATGTCCTAGCAATGTAATCGTCTATGACGTC

Table S2 - Sequence of the oligonucleotides used for the plasmid and strain construction.

**Name**	Sequence (5’ to 3’)
	**Construction for the plasmid pFB-Rad50 carrying the *rad50-C47* or *-C126* mutation**<br>Underlined sequences indicate restriction enzyme cleavage sites.
KS3723	CGCGACGACTGGCAGAAGAGTCAGATCG
KS3724	ACTCTTCTGCCAGTCGTCGCGCTTGACC
KS3729	CGCCAGCGCAGTCAGATCGAGTGGGTG
KS3730	CTGACTGCGCTGGCGGTCGTCGCGCTT

KS3731	CATCGGGCGCGGATCC
KS3732	TGCAATAAACAAGTTAACAAC
	**Construction for the IR-cassettes**<br>Sequences in bold indicate the 32 bp inverted repeat sequence.
KS2181	AAACTCGAGCCTGAGTGCATTTGCAACATG
KS2182	AAAGAATTCCAATTGGAGCAATGAACCCAATAACGAAATC
kS3845	CACGAATTCGGGTACCGTTGATATTGCAAAGAGTGATAAAGGAACCCAATAAC GAAATC
KS3870	**CTTTATCACTCTTTGCAATATCAACGGTACCC**G
KS3871	AATTC**GGGTACCGTTGATATTGCAAAGAGTGATAAAG**
KS3872	AAATCTTTATCACTCTTTGCAATATC
KS3873	TAAGAATTCCAAAGAACCAAGGGGATTATTGATGCTTGCTGGAGCAATGAACC CAATAACG
	***rad1* disruption**
KS865	GGACGAGTAAACTTTTGTCTGCGTGGCGCATAGGAGAGGAGAGAGCACAGGA AACAGCTATGACC
KS866	AGCGTTATCATCAGTGGTCTTACCAGGAGATTCAAGATTTTCATCTGTTGTAAA ACGACGGCCAGT
	***rad50* mutation integration**
KS3511	CATCAACGATATCGATTCTAGAG
KS3522	CGTGCTTCTAACTCCTTTACTC
KS3548	GAGAGGACGATGTTCCGC
X022	CGAGCTCGAATTCATCGATAATACGACTCACTATAGGGCGA
	***MRE11-HA* strain construction**
KS1013	CCAAAGACGGATATTCTTGGAAGTCTCCTTGCTAAGAAAAGAAAACGTACGCTGCAGGT CGAC
KS1014	CTTGGTTATAAATAGGATATAATATAATATAGGGATCAAGTACAAATCGATGAATTCGA GCTCG

	***HDF2-HA* strain construction**
KS865	GGACGAGTAAACTTTTGTCTGCGTGGCGCATAGGAGAGGAGAG AGCACAGGAAACAGCTATGACC
KS866	AGCGTTATCATCAGTGGTCTTACCAGGAGATTCAAGATTTTCATC TGTTGTAAAACGACGGCCAGT

Table S3 – Strains used in this study.

**Strain**	**Genotype**	
KSC2244	*MATainc ade1 his2 leu2 trp1 ura3 mec1Δ::LEU2 rad50Δ::NatMX sml1Δ::LEU2* [pRS426-RAD50],	**Screening**
KSC1560	*MATainc ade1 his2 leu2 trp1 ura3 sml1Δ::LEU2 ADH4cs::HIS2*	**Fig. 1C, 1D**
KSC1561	KSC1560 with *mec1Δ::LEU2*	
KSC1700	KSC1560 with *mec1Δ::LEU2 sae2Δ::URA3*	
KSC2726	KSC1560 with *mec1Δ::LEU2 rad50S::URA3*	
KSC3937	KSC1560 with *mec1Δ::LEU2 rad50-C47::URA3*	
KSC3938	KSC1560 with *mec1Δ::LEU2 rad50-C126::URA3*	

KSC1516	*MATainc ade1 his2 leu2 trp1 ura3 ADH4cs::HIS2*	**Fig. 1G, 2B,**
KSC2192	KSC1516 with *rad50Δ::HphMX*	
KSC2726	KSC1516 with *rad50S::URA3*	
KSC3937	KSC1516 with *rad50-C47::URA3*	
KSC3938	KSC1516 with *rad50-C126::URA3*	
KSC4790	KSC1516 with *MRE11-3HA::KanMX*	**Fig. 1H**
KSC4795	KSC1516 with *rad50Δ::HphMX*	
KSC4791	KSC1516 with *rad50-C47::URA3*	
KSC4793	KSC1516 with *rad50-C126::URA3*	
NKY1551	*MATa/α ho::LYS2/", lys2/", ura3/", leu2::hisG/", his4B-LEU2(MluI)/his4X-LEU2(BamHI)-URA3, arg4-bgl/arg4-nsp*	**Fig. 2A, 2B, 4, 5**
MSY6011	NKY1551 with *rad50-C47::URA3*	
MSY6030	NKY1551 with *rad50-C126::URA3*	
MSY1758	NKY1551 with *rad50::URA3*	
MSY6844	NKY1551 with *exo1-D173A::Afl*I	
MSY6841	NKY1551 with *rad50-C47::URA3 exo1-D173A::Afl*I	
SLY19	*MATα::URA3-HOcs hoΔ, hmlΔ::ADE1, hmrΔ::ADE1, ade1-100, leu2-3,112, lys5, trp1::hisG, ura3-52, ade3::GAL::HO*	**Fig. 2C**
MSY6079	SLY19 with *rad50-C47::ura3::hphMX4*	
MSY6708	SLY19 with *rad50-C126::ura3::hphMX4*	

MSY6285	SLY19 with *rad50S (K81I)::Ssp*I	
DIY059	SLY19 with *sae2::hphMX4*	
MSY6126	SLY19 with *rad50-C47::ura3::hphMX4, sae2:: hphMX4*	
DIY059	SLY19 with *sae2:: hphMX4*	
MSY6126	SLY19 with *rad50-C47::ura3::hphMX4, sae2::hphMX4*	
MSY6690	*MATa*/alpha*, ho::LYS2/", lys2/", ura3/", leu2::hisG/", SPO11-3FLAG::KANMX*	**Fig. 6**
MSY6632	MSY6690 with *rad50-47::URA3, SPO11-3FLAG::KANMX*	
MSY6996	MSY6690 with *rad50S, SPO11-3FLAG::KANMX*	
KSC4515	*MATainc ade1 his2 leu2 trp1 ura3 mnt2::HIS2 HphMX-Δ5*	**Fig. 7**
KSC4621	KSC4515 with *KanMX-Δ3-IR4*	
KSC4622	KSC4515 with *KanMX-Δ3-IR4 rad50S::TRP1*	
KSC4623	KSC4515 with *KanMX-Δ3-IR4 rad50-C47::TRP1*	
KSC4631	KSC4515 with *KanMX-Δ3-IR17*	
KSC4633	KSC4515 with KanMX-Δ3-IR17 *rad50S::TRP1*	
KSC4632	KSC4515 with *KanMX-Δ3-IR17 rad50-C47::TRP1*	
KSC4642	KSC4515 with *KanMX-Δ3-IR0*	
KSC4643	KSC4515 with *KanMX-Δ3-IR0 rad50S::TRP1*	
KSC4644	KSC4515 with *KanMX-Δ3-IR0 rad50-C47::TRP1*	
KSC4677	KSC4515 with *KanMX-Δ3-IR100 rad1Δ::LEU2*	
KSC4678	KSC4515 with *KanMX-Δ3-IR100 rad1Δ::LEU2 rad50S::TRP1*	
KSC4679	KSC4515 with *KanMX-Δ3-IR100 rad1Δ::LEU2 rad50-C47::TRP1*	

**KSC4190**	*HDF2-6HA::TRP1 rad50Δ::URA3 VII-L::KanMX-HO*	**Fig. S8**
**KSC4191**	*HDF2-6HA::TRP1 VII-L::KanMX-HO*	
**KSC4473**	*HDF2-6HA::TRP1 rad50S::URA3 VII-L::KanMX-HO*	
**KSC4474**	*HDF2-6HA::TRP1 rad50-C47::URA3 VII-L::KanMX-HO*	
	All the strains are isogenic to KSC2217 (*MAT***a**-inc *ade1 his2 leu2 trp1 ura3 VII-L::KanMX-HO*)[1].	

Supplementary Methods

Chromatin immunoprecipitation (CHIP) assay to detect Ku binding at DNA ends

The *HDF2-HA* cells were generated by a PCR-base method [2] using the primer pair (KSC865 and KSC866). The CHIP assay was carried out and analyzed by using Bio-Rad CFX manager (1.5) [3].

Molecular weight measurement by mass photometry

Mass photometry measurements were performed on a 2MP-0132 mass photometer (Refeyn Ltd). Coverslips (No. 1.5 H thickness, 24 × 50 mm, VWR) were dipped in Milli-Q-water, isopropanol and Milli-Q-water, and dried under a stream of gaseous nitrogen. Silicone gaskets (CultureWellTM Reusable Gasket, Grace Bio-Labs) were then placed on the coverslips to create wells for sample loading. For the measurements, the well was filled with MP buffer (25 mM Tris–HCl pH 7.5, 150 mM NaCl) to focus the microscope onto the coverslip surface. For Mre11-Xrs2 (MX) and Rad50 variants alone, 3 µl of protein solution (1000 µM) was mixed into 17 µl of MP buffer (150 nM final) before the measurement. To measure the interaction between MX and the Rad50 variants, MX and Rad50 were incubated at 500 nM each for 5 min at room

temperature. After incubation, 6 µl of the mixture was added to the 14 µl droplet of MP buffer used for the focusing. The measurement was performed by recording a 1 minute-long movie using AcquireMP (Refeyn Ltd) software that was then analyzed using DiscoverMP (Refeyn Ltd). A molecular marker (NativeMarkTM Unstained Protein Standard, Invitrogen) was used as a reference to convert the measured optical reflection-interference contrast into a molecular mass.

Cytological analysis

Chromosome spreads were prepared by the Lipsol method and analyzed by immunostaining with guineapig anti-Rad51 or rabbit anti-Dmc1 antibodies [4].

Supplementary Figures

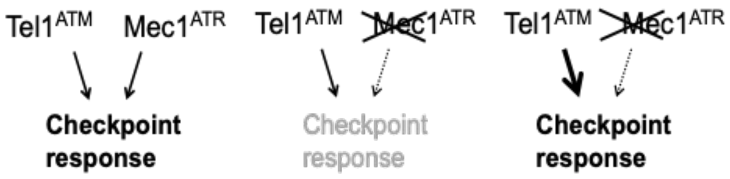

Fig. S1. Enhancement of the Tel1 signaling pathway restores *MEC1* loss-of-function.

See the result section for explanation.

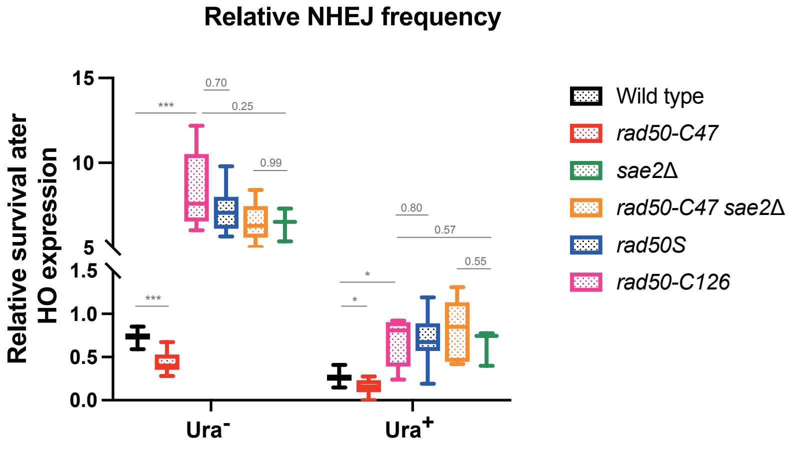

Fig. S2. NHEJ activity of *rad50* mutants. The generation of Ura^+ and Ura^- cells is shown separately.

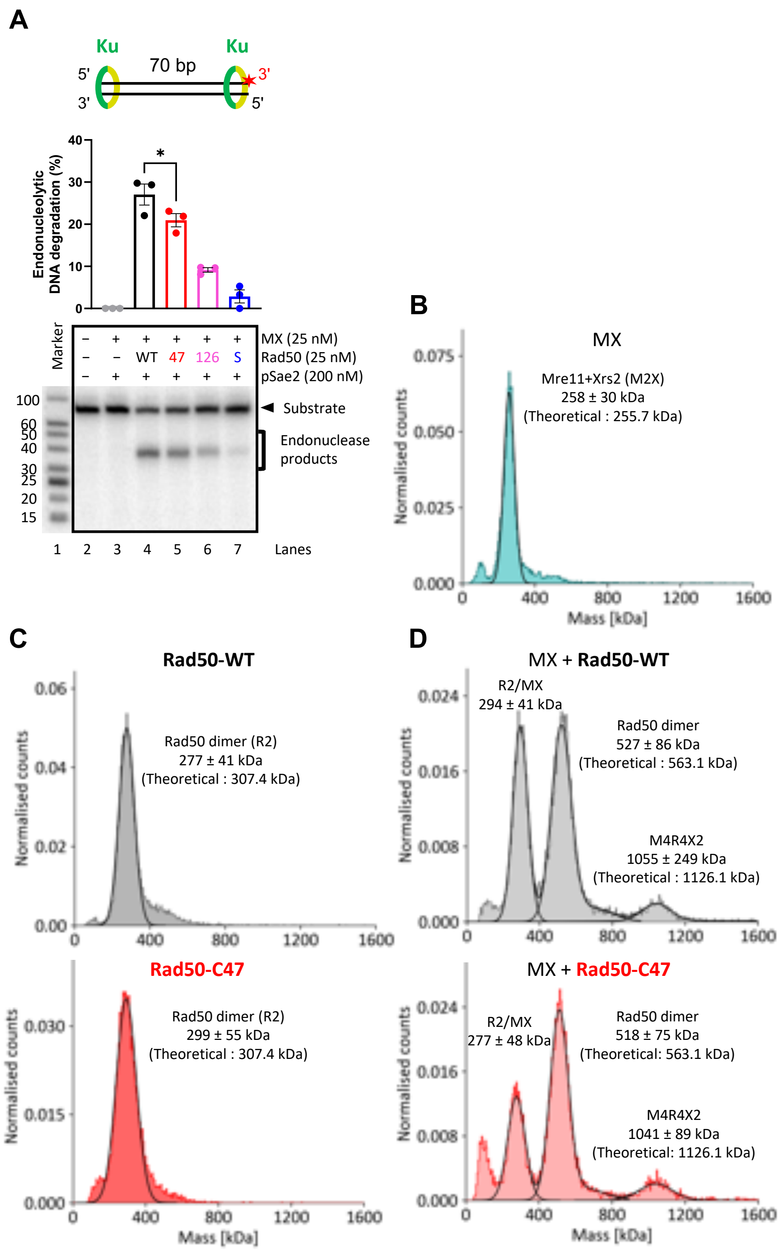

Fig. S3. Effect of the *rad50-C47 or rad50-C126* mutation on MRX-Sae2 endonuclease and MRX complex formation *in vitro*

A. Processing of Ku-blocked (10 nM) DNA substrate by MRX-Sae2 with Rad50 variants as indicated. Top: schematic representation of the DNA substrate. Middle: quantitation; error bars represent SEM; n=3. Bottom: a representative experiment. P <0.05 (*, 0.0261), two-tailed t-test.

B. Measured molecular weight distribution of Mre11-Xrs2 (150 nM) using mass photometry. The theoretical and measured (median ± SD) weights of M2X (2 Mre11 and 1 Xrs2 subunits) is indicated.

C. Measured molecular weight distributions of Rad50-WT (top) and -C47 (bottom) using mass photometry (150 nM). The theoretical and measured (median ± SD) weights of the Rad50 dimer (R2) is indicated.

D. Measured molecular weight distributions of MX and Rad50-WT (top) and Rad50-C47 (bottom) incubated for 5 min at room temperature (500 nM) and diluted to 150 nM each immediately before measurement. The theoretical and measured (median ± SD) weights of the MRX complex (M2R2X) and of a MRX dimer (M4R4X2) are indicated.

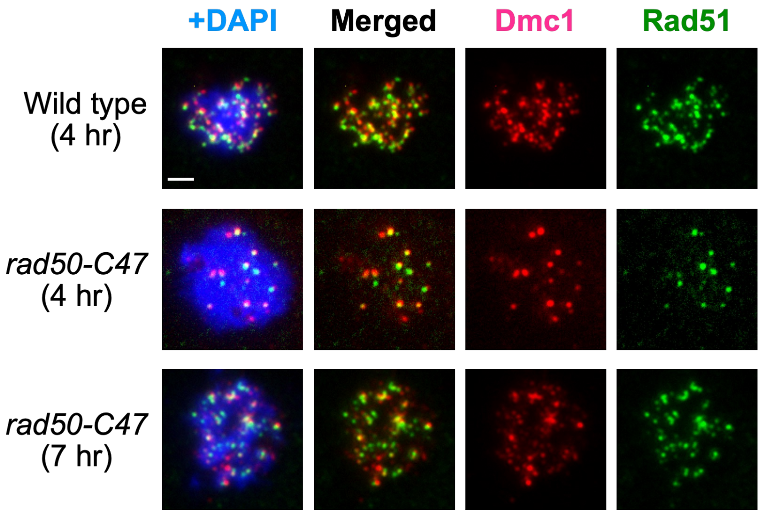

Fig. S4. Effect of the *rad50-C47* mutation on Dmc1 focus formation during meiosis.

Cells were treated and examined using anti-Dmc1 and anti-Rad51 antibodies as described in Fig. 5A.

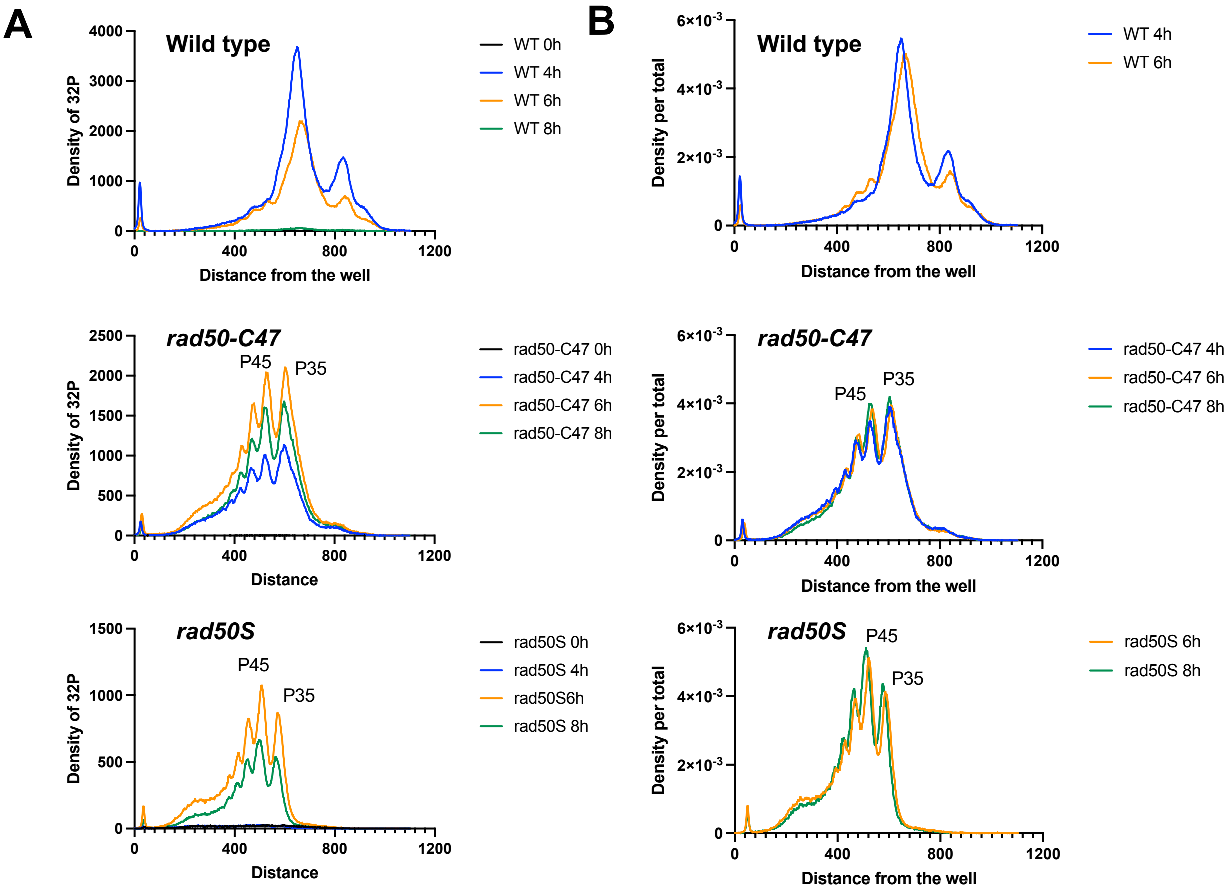

Fig. S5. The peak patterns in the time-course (Fig. 6D, left) were summarized in one graph for each strain.

A. Peak signals were plotted for each strain.

B. Peak signals divided by total signals were plotted for each strain.

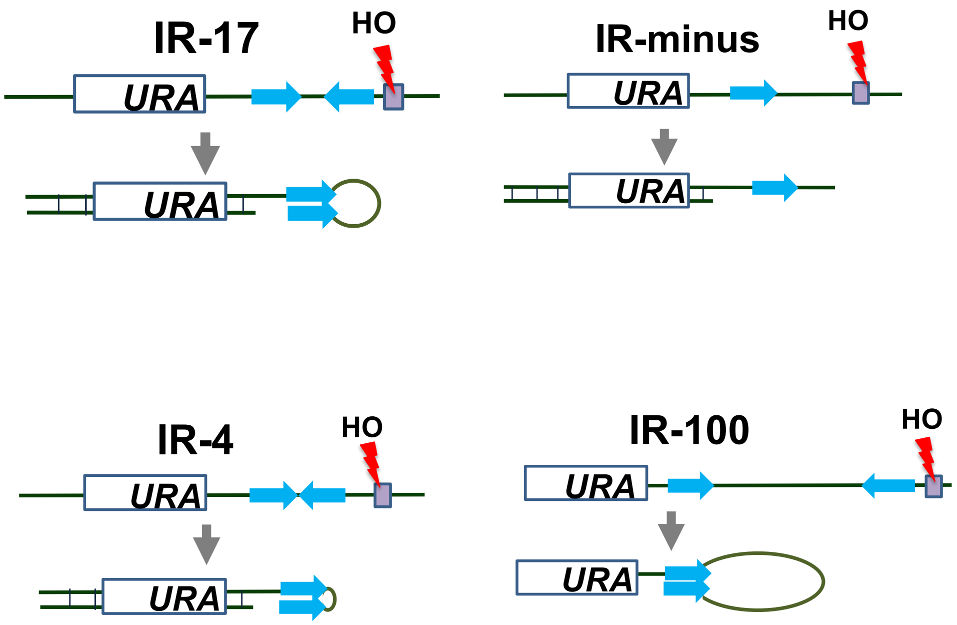

Fig. S6. Hairpin formation at IR cassettes after HO-induced DSB generation

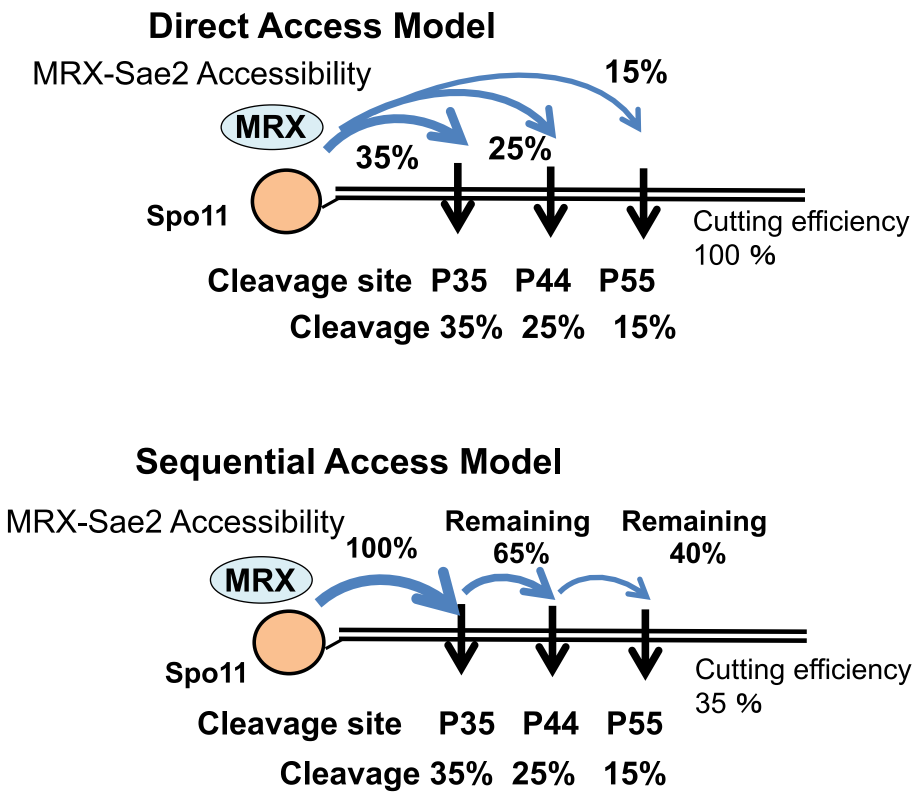

Fig. S7. Direct Access Model and Sequential Access Model

MRX-Sae2 may directly access the cleavage sites after recognizing Spo11 at DNA ends (Direct access model). In this case, MRX-Sae2 may access nearby cleavage sites more efficiently than distant ones. Alternatively, MRX-Sae2 may sequentially access the cleavage sites one by one after recognizing Spo11 at DNA ends. In this case, MRX-Sae2 excises at each cleavage site with low cleavage efficiency (35%).

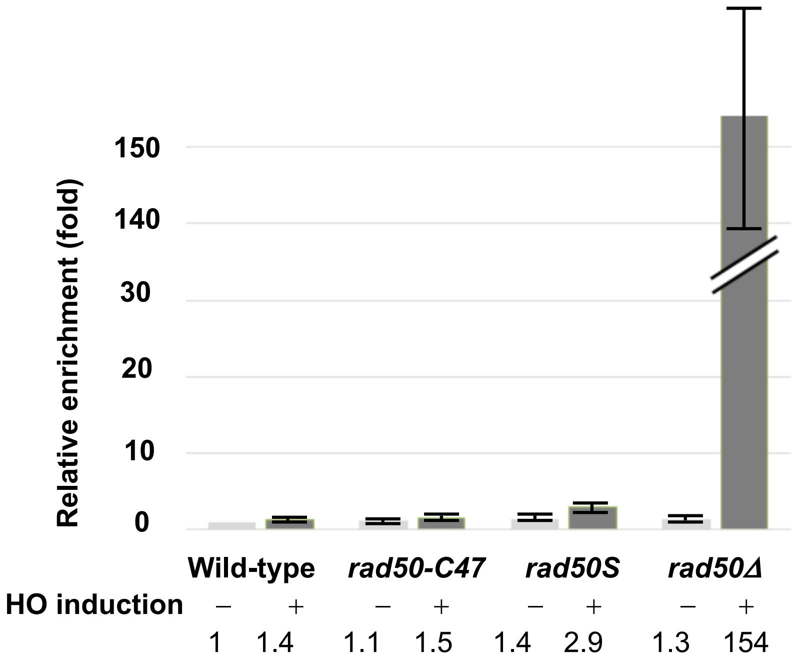

Fig. S8. **Effect of the *rad50-C47* mutation on Ku accumulation near a DSB end**

Cells expressing HA-tagged Hdf2 protein were subjected to chromatin immunoprecipitation before and after HO expression.

References

1. Nakada, D., Matsumoto, K., and Sugimoto, K. (2003). ATM-related Tel1 associates with double-strand breaks through an Xrs2-dependent mechanism. Genes & Dev. *17*, 1957-1962.
2. Janke, C., Magiera, M.M., Rathfelder, N., Taxis, C., Reber, S., Maekawa, H., Moreno-Borchart, A., Doenges, G., Schwob, E., Schiebel, E., and Knop, M. (2004). A versatile toolbox for PCR-based tagging of yeast genes: new fluorescent proteins, more markers and promoter substitution cassettes. Yeast *21*, 947-962. 10.1002/yea.1142.
3. Hirano, Y., Fukunaga, K., and Sugimoto, K. (2009). Rif1 and Rif2 inhibit localization of Tel1 to DNA ends. Mol. Cell *33*, 312-322.
4. Hayase, A., Takagi, M., Miyazaki, T., Oshiumi, H., Shinohara, M., and Shinohara, A. (2004). A protein complex containing Mei5 and Sae3 promotes the assembly of the meiosis-specific RecA homolog Dmc1. Cell *119*, 927-940. 10.1016/j.cell.2004.10.031.
