# Supplementary material for: Sae2 controls Mre11 endo- and exonuclease activities by different mechanisms
Source: Nat Commun. 2024 Aug 22;15:7221. doi: 10.1038/s41467-024-51493-5 (PMC11341764; doi:10.1038/s41467-024-51493-5)
Supplement: Supplementary file 3 — Reporting Summary [file 41467_2024_51493_MOESM3_ESM.pdf]

nature portfolio

Corresponding author(s): Katsunori Sugimoto

Last updated by author(s): Jul 2, 2024

Reporting Summary

Nature Portfolio wishes to improve the reproducibility of the work that we publish. This form provides structure for consistency and transparency in reporting. For further information on Nature Portfolio policies, see our Editorial Policies and the Editorial Policy Checklist.

Statistics

For all statistical analyses, confirm that the following items are present in the figure legend, table legend, main text, or Methods section.

n/a	Confirmed	
☐	☒	The exact sample size (n) for each experimental group/condition, given as a discrete number and unit of measurement
☒	☐	A statement on whether measurements were taken from distinct samples or whether the same sample was measured repeatedly
☐	☒	The statistical test(s) used AND whether they are one- or two-sided<br>*Only common tests should be described solely by name; describe more complex techniques in the Methods section.*
☒	☐	A description of all covariates tested
☒	☐	A description of any assumptions or corrections, such as tests of normality and adjustment for multiple comparisons
☐	☒	A full description of the statistical parameters including central tendency (e.g. means) or other basic estimates (e.g. regression coefficient) AND variation (e.g. standard deviation) or associated estimates of uncertainty (e.g. confidence intervals)
☐	☒	For null hypothesis testing, the test statistic (e.g. F, t, r) with confidence intervals, effect sizes, degrees of freedom and P value noted<br>*Give P values as exact values whenever suitable.*
☒	☐	For Bayesian analysis, information on the choice of priors and Markov chain Monte Carlo settings
☒	☐	For hierarchical and complex designs, identification of the appropriate level for tests and full reporting of outcomes
☒	☐	Estimates of effect sizes (e.g. Cohen's d, Pearson's r), indicating how they were calculated

Our web collection on statistics for biologists contains articles on many of the points above.

Software and code

Policy information about availability of computer code

Data collection	Gels were acquired using a Typhoon Phosphor Imager FLA 9500 (version 1.0) and photo scanner operated with CanoScan 9000F Mark II scanner and ImageCapture v6.6(525) software. Blots were captured using Fusion FX7 capture software (Vilber Imaging, Version FX7 Edge 18.12 -SN). Mass photometry data was acquired with AcquireMP software (Refeyn Ltd, Version AcquireMP 2023 R1.1). Real time PCR data were collected by Bio-Rad CFX manager (1.5)
Data analysis	Gels were analyzed using ImageJ 1.53g and the results were plotted using Graph Pad Prism 10.2.2 (397). Mass photometry data was analyzed using DiscoverMP software (Refeyn Ltd, Version v2023 R1.2).<br>ChIP data was analyzed by Bio-Rad CFX manager (1.5).

For manuscripts utilizing custom algorithms or software that are central to the research but not yet described in published literature, software must be made available to editors and reviewers. We strongly encourage code deposition in a community repository (e.g. GitHub). See the Nature Portfolio guidelines for submitting code & software for further information.

Data

Policy information about availability of data

All manuscripts must include a data availability statement. This statement should provide the following information, where applicable:

- Accession codes, unique identifiers, or web links for publicly available datasets
- A description of any restrictions on data availability
- For clinical datasets or third party data, please ensure that the statement adheres to our policy

All the raw data are provided in the source data file.

Research involving human participants, their data, or biological material

Policy information about studies with human participants or human data. See also policy information about sex, gender (identity/presentation), and sexual orientation and race, ethnicity and racism.

Reporting on sex and gender	N/A
Reporting on race, ethnicity, or other socially relevant groupings	N/A
Population characteristics	N/A
Recruitment	N/A
Ethics oversight	N/A

Note that full information on the approval of the study protocol must also be provided in the manuscript.

Field-specific reporting

Please select the one below that is the best fit for your research. If you are not sure, read the appropriate sections before making your selection.

☒ Life sciences ☐ Behavioural & social sciences ☐ Ecological, evolutionary & environmental sciences

For a reference copy of the document with all sections, see nature.com/documents/nr-reporting-summary-flat.pdf

Life sciences study design

All studies must disclose on these points even when the disclosure is negative.

Sample size	Sample-size calculation was not applied
Data exclusions	None
Replication	All experimental data shown in Fig. 2A, C, Fig. 4C, D, Fig. 5, Fig. 6C were summarized from at least three independent trials. The histogram shown in Fig. 6D is from a representative image shown in Fig. 6D after multiple trials. The data was backed up by examining phenotypes of rad50 deletion mutants carrying the respective rad50 mutation (Fig. 7). The experiments were repeated multiple times, as indicated (Fig. 3).
Randomization	Not required for this work
Blinding	Not required for this work

Reporting for specific materials, systems and methods

We require information from authors about some types of materials, experimental systems and methods used in many studies. Here, indicate whether each material, system or method listed is relevant to your study. If you are not sure if a list item applies to your research, read the appropriate section before selecting a response.

Materials & experimental systems

n/a	Involved in the study	
☐	☒	Antibodies
☐	☒	Eukaryotic cell lines
☒	☐	Palaeontology and archaeology
☒	☐	Animals and other organisms
☒	☐	Clinical data
☒	☐	Dual use research of concern
☒	☐	Plants

Methods

n/a	Involved in the study	
☒	☐	ChIP-seq
☒	☐	Flow cytometry
☒	☐	MRI-based neuroimaging

Antibodies

Antibodies used

anti-Pgk1: Abcam ab113687, Mouse monoclonal [22C5D8]
anti-Rad51: outsourced to MBL Co Ltd, Ginea pig polyclonal, ref: Shinohara et al., Nat Genet, 2008
anti-Mre11: outsourced to MBL Co Ltd, Rabbit polyclonal, ref: Shinohara et al., Frontiers in Cell and Developmental Biology, 2023
anti-Dmc1: homemade, Rabbit polyclonal, ref: Hayase et al., Cell, 2004
anti-DYKDDDK tag: FUJIFILM Wako 014-22383, Mouse monoclonal [1E6]
Anti-FLAG M2 antibody (F3165, Sigma),
Anti-HA (16B12), Biolegend
Anti-Rad50: gift from John Petrini

Validation

anti-Pgk1: Abcam
anti-Rad51: Shinohara et al., Nat Genet, 2008
anti-Mre11: Shinohara et al., Frontiers in Cell and Developmental Biology, 2023
anti-Dmc1: Hayase et al., Cell, 2004
anti-DYKDDDK tag: FUJIFILM Wako
Anti-FLAG M2 antibody, Sigma
Anti-HA (16B12), Biolegend
Anti-Rad50: Park et al. 2013 (PMID:28134932), gift from John Petrini

Eukaryotic cell lines

Policy information about cell lines and Sex and Gender in Research

Cell line source(s)

All yeast strains used in Fig. 2, 4, 5 and 6 will be deposited in National BioResource Project - Yeast (Japan) :https://yeast.nig.ac.jp/All other strains will be deposited at public sources.
We will provide listed stocks upon request until available from public resources.

Authentication

Describe the authentication procedures for each cell line used OR declare that none of the cell lines used were authenticated.

Mycoplasma contamination

Confirm that all cell lines tested negative for mycoplasma contamination OR describe the results of the testing for mycoplasma contamination OR declare that the cell lines were not tested for mycoplasma contamination.

Commonly misidentified lines
(See ICLAC register)

Name any commonly misidentified cell lines used in the study and provide a rationale for their use.

Plants

Seed stocks

N/A

Novel plant genotypes

N/A

Authentication

N/A
